# Experiences of Telehealth Reimbursement Policies in Federally Qualified Health Centers

**DOI:** 10.1001/jamanetworkopen.2024.59554

**Published:** 2025-02-12

**Authors:** Thalia Porteny, Sorcha A. Brophy, Emily Burroughs

**Affiliations:** 1 Department of Health Policy and Management, Mailman School of Public Health, Columbia University, New York, New York; 2 Robert N. Butler Columbia Aging Center, Mailman School of Public Health, Columbia University, New York, New York; 3 Department of Sociomedical Sciences, Mailman School of Public Health, Columbia University, New York, New York

## Abstract

**Importance:**

The impact of Medicaid telehealth reimbursement policies on staffing and patient-centered care in the safety net are largely unknown but critical to successful and equitable access to telehealth.

**Objective:**

To identify and characterize federally qualified health center (FQHC) staff and leadership’s perceptions of the benefits and drawbacks of Medicaid telehealth reimbursement policies.

**Design, Setting, and Participants:**

This qualitative study used semistructured interviews conducted from April 2022 to January 2024 with snowballed sampled participants consisting of FQHC leadership, clinicians, and administrative staff in 6 FQHCs representing the 5 boroughs in New York, New York.

**Main Outcomes and Measures:**

Participants described telehealth experiences, including factors impending or contributing to staff turnover, patient satisfaction, and financial sustainability within FQHCs. Thematic analysis was used to analyze the data.

**Results:**

Of 56 interviews, 26 participants (46.4%) were part of the leadership team, 18 (32.1%) were clinical staff, 8 (14.3%) were program support staff, 7 (12.5%) were enabling services staff, 3 (5.4%) were site directors, and 3 (5.4%) were another staff category. Three overarching themes characterized staff and leadership understanding of the impact of Medicaid telehealth reimbursement policies on FQHCs: (1) Medicaid telehealth policy design was perceived to exacerbate a workforce shortage, particularly among mental health care practitioners; (2) patients ranged in preferences and ability to access telehealth while FQHCs struggled to attain resources for telehealth; and (3) FQHC leadership envisioned a productive hybrid model where telehealth complements on-site care. FQHC staff and leadership reported opportunities to improve compliance, no-shows, and workflows through telehealth, but improvements in funding policy, such as payment parity and more grants that can be used to address telehealth infrastructure (eg, Internet access, equipment, and literacy), are urgently needed.

**Conclusions and Relevance:**

In this qualitative study, staff at FQHCs perceived the current telehealth Medicaid reimbursement policies in New York State as a factor that exacerbated inequities to accessing care, particularly for mental health needs. These findings indicate that although telehealth brings new opportunities to advance patient-centered care, there are serious challenges on the path toward equitable care because telehealth is not yet integrated into payment in a sustainable way.

## Key Points

**Question:**

How are Medicaid telehealth reimbursement policies perceived by federally qualified health center (FQHC) leadership and staff?

**Findings:**

In this qualitative study involving 56 interviews conducted at 6 FQHCs in New York, New York, between April 2022 and January 2024, participants perceived an association between low Medicaid telehealth reimbursement and a workforce crisis in FQHCs, particularly among mental health care practitioners.

**Meaning:**

These findings suggest that current Medicaid reimbursement policies in New York State may exacerbate inequities to access care, particularly for mental health needs.

## Teaser

This qualitative study examines perceptions of the benefits and drawbacks of Medicaid telehealth reimbursement policies among staff and leadership at federally qualified health centers in New York, New York.

## Introduction

Telehealth is an increasingly accepted way to deliver clinical health care with demonstrated benefits to improved access to mental health and managed care.^1,2,3,4^ However, telehealth may also exacerbate existing health disparities because access requires broadband, digital literacy, and devices, all of which are associated with age, race, English proficiency, socioeconomic levels, and other related factors linked to the structural determinants of health.^5,6,7,8,9^ Moreover, as telehealth technology advances and uptake increases, quality-care inequities are emerging between those who are able to access care through video-based platforms vs audio only, as well as between insurance types.^10,11^ Some insurance plans do not reimburse the same amount for services provided via telehealth vs in-person but others do.^12,13,14,15^ These gaps introduce questions about how to make telehealth work in a manner that improves health equity.^16^

Although it does not respond to all root causes of telehealth inequity, one policy lever that can promote improved access to telehealth is payment parity (ie, reimbursing the same amount for a service provided via telehealth vs in-person).^17^ One reason is that payment parity incentivizes practitioners with preferences to work remotely to provide care to many patients who would otherwise face barriers to care and consequently increase utilization.^12,18^ This option may be particularly important for safety-net health care facilities, which face additional challenges recruiting and retaining practitioners.^19^ Remote visits also provide needed flexibility for patients who face challenges scheduling visits around work or caregiving responsibilities or who lack the ability to travel to see a practitioner.^20,21^ During the COVID-19 pandemic, Medicaid expanded provisions and offered parity payments for telehealth visits.^12^ As the COVID-19 Public Health Emergency (PHE) came to an end, some states maintained Medicaid telehealth reimbursement parity, but others rolled back all or some of their telehealth flexibilities.^22,23^ However, little is known about whether and how rescinding Medicaid telehealth reimbursement may be affecting practitioner networks with a large share of Medicaid enrollees.

Federally qualified health centers (FQHCs) rely heavily on Medicaid to pay practitioners to provide low-cost primary care services to approximately 1 in 12 people needing mental and physical health care.^24^ However, FQHCs are currently experiencing a workforce crisis for reasons that are incompletely understood. According to a recent report,^25^ in mid-2022, approximately 68% of FQHCs reported having lost 5% to 25% of their workforce in the prior 6 months, and 15% of community health centers (CHCs) reported having lost 25% to 50% of their workforce. This problem is particularly pronounced in urban health centers, which report higher rates of attrition than rural CHCs.^25^ CHCs report increasing workforce shortages and high turnover due to noncompetitive wages, low motivation, and burnout.^25,26^ Studies on the impacts of telehealth on practitioner burnout are mixed. One study^27^ found that psychiatrists experienced additional mental fatigue focusing on nonverbal communication and building rapport with patients via telehealth. Meanwhile, studies^28,29,30,31^ have also shown that successfully implemented telehealth in community-based primary care settings can reduce practitioner burnout through increased workflow efficiency, including reduced no-show rates. Moreover, there is limited knowledge of the possible link between the FQHC workforce shortage and changes in Medicaid telehealth reimbursement.^32^

FQHCs in New York State have one of the highest percentages (49%) of Medicaid revenue dependency in the US.^33^ After the COVID-19 PHE, New York State legislated Medicaid parity payments for video-based and audio telehealth services.^33,34^ The latter proved beneficial to marginalized patients (eg, older, limited English proficiency, or limited digital literacy).^35^ However, New York State instituted a different fee for in-person vs off-site services. For hospitals and practitioners that charge facility fees, the changes between billing for telehealth vs in person were marginal, but for nearly all FQHCs, which bill a bundled prospective payment system (PPS) rate, the difference between billing for in-person services vs off-site is approximately one-third of the amount. FQHCs can bill the PPS in-person amount for telehealth, but either the practitioner or the patient must come into the facility for the appointment. Notably, this payment differential is exempted for practitioners licensed as Article 31 or 32; however, most FQHCs deliver behavioral health services in the Article 28 setting.^36^ Moreover, FQHCs are obligated to deliver the same services, including those via telehealth, that a Medicaid recipient would receive for uninsured individuals to maintain their ability to bill the bundled PPS rate.^37^ However, the effects of this payment differential in New York State remain incompletely understood.

Most studies investigating telehealth utilization use surveys or claims data, which, although useful in evidencing disparities, are insufficient for understanding how organizations navigated shifting Medicaid reimbursement policies.^7,30,38^ This qualitative study conducted at 6 urban FQHC sites uniquely addresses these gaps by analyzing the perception of recent impacts of Medicaid telehealth reimbursement policies on staffing and how these might affect patient-centered care in New York State from the FQHC perspective.

## Methods

This qualitative study was approved by the institutional review board at Columbia University Medical Center. Interviews were conducted via online video conferencing and in person from April 2022 to January 2024. The study goals and rationale were explained, and participants provided verbal informed consent. This study followed the Consolidated Criteria for Reporting Qualitative Research (COREQ) reporting guideline.

### Data Collection

A social scientist with expertise in qualitative methods and organizational sociology (S.A.B.) designed the interview guides on the basis of a literature review with input from a primary care clinician with experience working community-based settings (not one of the authors of this article). The guides were refined by the research team. Informed by qualitative interviewing methodological literature,^39^ our interview guide focused on several key areas, including financing, reimbursement policies, health equity, workforce challenges, and patient experiences. Interview questions were tailored according to interviewees’ position.

The study team selected a purposeful sample of 6 CHC sites, conducting initial outreach using email contact information for FQHC leadership in New York, New York. The selected sites provide geographic variation across all 5 boroughs and serve diverse patient populations. These CHCs range from 2 to 13 sites, representing both small and large operations. By use of a snowball sampling approach to recruitment, these contacts referred other relevant employees within each site.^40^ The study team continued recruitment at individual and site levels until reaching site and thematic saturation.^41^

At each site, the study team interviewed CHC leadership team members, (ie, Chief Executive Officers, Chief Operations Officers, Chief Financial Officers, and Chief Medical Officers) and site-level leadership (ie, site directors). Additionally, we spoke with clinical staff (eg, nurse practitioners and dental hygienists), enabling services staff (eg, case managers and patient navigators), and program support staff (eg, outreach and grant coordinators). No demographic data were collected on interviewees owing to privacy protection.

### Statistical Analysis

The study team produced verbatim transcripts from audio recordings of each interview. The team developed a preliminary codebook employing an abductive approach,^42^ iterating between emerging findings and existing research about structural limitations in health care delivery, policy design, and health disparities. Codes selected for the codebook represent key concepts and are used to organize qualitative data using NVivo 14 qualitative coding software.^43^ All 3 authors of this article independently coded an initial set of 8 interview transcripts to assess interrater reliability and further refine the codebook. Iterative deliberation produced a consensus about coding discrepancies and emergent codes. The team refined the codebook and coded the full dataset accordingly, categorizing data into themes and subthemes. We further analyzed these themes by running queries of specific codes in NVivo, identifying the most salient themes, as well as convergent and divergent themes in the data.

## Results

The study team conducted 56 semistructured interviews. Of 56 interviews, 26 participants (46.4%) were part of the leadership team, 18 (32.1%) were clinical staff, 8 (14.3%) were program support staff, 7 (12.5%) were enabling services staff, 3 (5.4%) were site directors, and 3 (5.4%) were another staff category (Table 1). The findings indicate that although telehealth brings new opportunities to advance patient-centered care, there are serious challenges on the path toward equitable care because telehealth is not yet integrated into payment in a sustainable way. Three overarching themes characterized the impact of Medicaid telehealth reimbursement policies on FQHCs: (1) Medicaid telehealth policy design was perceived to exacerbate a workforce shortage, particularly among mental health care practitioners; (2) patients ranged in preferences and ability to access telehealth while FQHCs struggled to attain resources for telehealth; and (3) FQHC leadership envisioned a productive hybrid model where telehealth complements on-site care (Table 2).

**Table 1.** Key Informant Characteristics

Characteristic	Respondents, No. (%) (N = 56)
Community health center	
1	11 (19.6)
2	6 (10.7)
3	10 (17.8)
4	3 (5.4)
5	8 (14.2)
6	16 (28.6)
Position type^a^	
Federally qualified health center–wide leadership team^b^	26 (46.4)
Site directors	3 (5.4)
Clinical staff^c^	18 (32.1)
Program support staff^d^	8 (14.3)
Enabling services staff^e^	7 (12.5)
Other	3 (5.4)

^a^ Several key informants filled multiple position types.

^b^ Leadership teams include individuals in senior roles who provide leadership across all sites of the federally qualified health center. This includes c-suite leaders (eg, Chief Executive Officers, Chief Medical Officers, Chief Operating Officers, and Chief Financial Officers), as well as roles such as directors of government affairs, policy, marketing, and communication.

^c^ We interviewed select clinical staff, including nurse practitioners, dentists, hygienists, and nutritionists to gain an understanding of on-the-ground challenges related to implementation.

^d^ Program support staff include individuals in administrative roles such as grant managers.

^e^ Enabling services staff are nonclinical staff who engage with patients to facilitate access to care and improve health outcomes. These include health home staff, patient navigators, and peer coaches.

**Table 2.** Themes and Example Quotations

Theme	Quotation
1: Medicaid telehealth policy design was perceived to exacerbate a workforce shortage, particularly among mental health practitioners	“The plan is there, the bill is set, it’s promoted by the health chairs of the Assembly and Senate, like, all it needs to do is make it over the finish line.” “We’re [very upset] about the special treatment Article 31s are getting in general, in certain cases for like telehealth…that’s a big issue for us in terms of workforce. The biggest change that I’ve witnessed post public health emergency, is the telehealth piece.” “We had a candidate who was a nurse practitioner that said, no negotiation, she wants $185,000. That was what I was paying MDs…. So salary is key number one, hybrid work from home schedules is top number two.” “We have a limited amount of space. So a lot of our decisions for behavioral health are really around work space availability, because right now New York State is the only state that requires clinicians be in office. Everyone else still goes home to home. So we had to really do a lot of decision making around that.” “We attract those who are just qualified, we spend an entire year doing a really detailed training and guess what? After that the hospital comes and just doubles [their salary]…and we can’t compete.”
2: Patients ranged in preferences and ability to access telehealth while FQHCs struggled to attain resources for telehealth	“It’s more on the patient side, you know, to be able to embrace the technology, have the capability, afford the capability to have internet at home.... So those are the challenges….” “We struggle with telehealth with [the older adult] population.” “We need to be able to contribute to help them get better internet...I know it was a huge push in the beginning of the pandemic, but it went away....” “Sometimes our patients don’t want virtual. Some people, especially older populations, would prefer to come on-site or prefer to have an in-person interaction.”
3: FQHC leadership envision a productive hybrid model where telehealth complements on-site care	“At home you have a lot more flexibility. And you know, you don’t have to deal with the politics of space or anything like that.” “The [company service] are a telehealth LPN or RN nurse who is able to communicate with patients who are Spanish speaking, or Mandarin speaking…. They provide those services for predominantly Medicare or dual eligible patients who have multiple chronicity such as diabetes, hypertension, and sometimes behavioral health.” “[Patients] keep saying behavioral health needs to stay remote. [They] love having that. They will say, for medical, ‘I want it in person.’ But we do have, for those who are sitting in the sites, if someone comes in and says, ‘I want in-person,’ then we will provide it for them.” “Patients like telehealth, maybe not for everything, but certainly for some things, and to have the opportunity to choose to receive care the way that best suits your need on a given day is as important and is something that now health centers and their patients can’t walk away from.”

Abbreviations: FQHC, federally qualified health center; LPN, licensed practical nurse; RN, registered nurse.

### Theme 1: Medicaid Telehealth Policy Design Was Perceived to Exacerbate a Workforce Shortage, Particularly Among Mental Health Care Practitioners

Respondents often used Medicaid telehealth policy design as an example of how policymakers prioritized other institutions—such as hospitals and specialized mental health services with Medicaid beneficiaries—over CHCs. As one noted, “When a lot of the rules are made, or when a lot of the emergency fundings for [telehealth] programs come out, they’re all geared through the hospital, and then they expect the hospitals to work with everybody else, where we all know nine times out of 10, that doesn’t happen.... The decision makers at the top who pull the purse strings…are leaning towards hospitals.” Several interviewees noted an inconsistency with the exemption granted to the Office of Mental Health and the Office of Addiction Services Licensed under Articles 31 and 32 and with how FQHCs were reimbursed. FQHC leadership interpreted the exemption as unfair and unequal treatment from policymakers because Article 31 and 32 institutions were receiving higher telehealth reimbursement rates and could pay clinicians more for some of the same services FQHCs offer. One participant explained that, “[The] ambulatory patient group rate [is] a bundled rate just like ours. They don’t have the [off-site] distinction. They’re excluded. And so we’re saying, you treat that bundled rate that way. We have a bundled rate. Treat all the bundled rates the same and fix this for health centers.”

FQHCs consistently faulted Medicaid reimbursement policies for the severe workforce turnover of mental health practitioners, because these individuals were not willing to give up remote work after the PHE ended and found employment elsewhere. One participant said, “We lost 40% of our staff of therapists and psychiatrists the second we forced them to come in…to do telehealth in a building rather than their own home.” FQHCs also argued that the consequential loss of staff gravely affected patient access. One FQHC informed us that they had 700 patients on a waiting list for behavioral health services, because their health center lost half its behavioral health practitioners when they began to require that their practitioners work in the office, rather than remotely (they went from 8 practitioners to 4). Multiple FQHC leadership explained that they could not compete with the higher salaries and remote opportunities offered by non-FQHCs. As one explained, “Well, they can get paid more going someplace else, sitting in their house, where they can have that luxury.”

### Theme 2: Patients Ranged in Preferences and Ability to Access Telehealth While FQHCs Struggled to Attain Resources for Telehealth

According to participants, many patients experienced barriers to telehealth related to the social determinants of health, such as accessing internet and navigating new technology (eg, technology literacy), which were unaddressed by Medicaid reimbursement policies (Table 2). Some older adults seemed to have trouble accessing telehealth and required additional resources, which created extra work for health centers. One participant stated, “Some of the older population, [we] were doing extra check-ins to see if they had anyone in the house that could help them do [telehealth].” Some participants perceived FQHCs had a responsibility to improve telehealth access for their patients but were limited by funding opportunities. One noted that there were grant opportunities to help patients get internet access at the beginning of the pandemic, but these opportunities then disappeared. Others applied for funds but were unsuccessful.

The FQHCs in our study reported a range of patient preferences about modality of care; although some preferred virtual, others preferred on-site visits. Others sensed that, for some, modality preferences might be related to age: “I think it is also generational. The older folks don’t like it. They don’t like doing it…. So the older folks like telephonic, the younger folks do the telemedicine video visits much better.” On the other hand, one participant noted the additional flexibility telehealth afforded patients, noting that a “significant number of patients like having [telehealth] because it means they don’t have to commute every week to get to the appointment, they can have it at home. So it’s been a real big game changer for a lot of our patients….”

### Theme 3: FQHC Leadership Envisioned a Productive Hybrid Model Where Telehealth Complements On-Site Care

FQHC leadership embraced telehealth to improve workflows and mental health care access but participants stressed the importance of preserving in-person clinical care. Their rationale for using telehealth anchored on concerns about limited space in the health center facilities, greater scheduling flexibility for practitioners, and the perceived effectiveness of telehealth for mental health. One FQHC leader said, “We’re desperately in need of space…. We are literally sitting on top of each other. I made [the] decision to do a hybrid model primarily because of that.” Importantly, interviewees described telehealth as a tool to be used in limited ways—for instance, not for diagnosing physical health conditions. One clinician expressed, “[Telehealth] is great for like follow up on labs.... But if I must diagnose and treat off of a computer screen that terrifies me. I’m just not that confident in my skillset to do it without touching and listening and feeling.” In contrast, participants expressed greater enthusiasm for remote mental health care. One participant explained how making mental health practitioners come to the FQHC not only hindered workforce flexibility but did not add clinical value: “Every therapist…and psychiatrist [is] making financial sacrifices to work for folks like us, [and now] they have to come to the health center to get on the phone basically, and talk to their patients. And there is zero clinical value to that.”

Participants held a common belief that if telehealth reimbursement policies were well aligned with practitioners’ compensation expectations, as was the case during the COVID-19 PHE response, access and compliance issues would greatly improve in FQHCs because there would be more opportunities and flexibility to see practitioners. As one participant described, “Behavioral health compliance went up dramatically [during COVID-19]. Behavioral health was always an area where patients used to cancel or no show. Well, once you had a telemedicine platform for behavioral health, suddenly we had 100% compliance rate.” Another explained, “We used to have a 30% no show, but because of [telehealth during COVID-19] our no-show rates were reduced to like 16%, you know, so it got cut in half.”

## Discussion

This multisite qualitative study of Medicaid telehealth policy experiences among urban FQHCs in New York, New York, identified a perceived association between low Medicaid telehealth reimbursement and a workforce crisis, particularly among mental health care practitioners. The challenges that arose from Medicaid telehealth policy design and other funding mechanisms compounded existing barriers to telehealth access among safety-net health care users. Our findings also underscore the benefits of telehealth and its potential, including improving workflows, space utilization, and flexibility for both practitioners and patients. Still, participants cautioned against using only telehealth to manage their complex panel of patients, many of whom have complex comorbid conditions, and to consider the spectrum of patient modality preferences.

FQHCs are currently experiencing tremendous workforce attrition and challenges recruiting. The challenge is especially acute for behavioral health.^36,44^ Our findings help explain these declines. Unable to offer competitive salaries or flexibility to work remotely, FQHC leadership stressed they lacked the capacity to compete for specialized staff, particularly mental health care practitioners. As a result, their patient population was losing access to care. To overcome this, participants suggested that legislation must be corrected to allow FQHCs to offer parity payments for telehealth services. If telehealth Medicaid reimbursement changes for FQHCs in New York State, future research must examine whether this shift resolves staff turnover and whether telehealth sustainably facilitates improvements in no-shows and compliance, or whether other contributing factors are the main drivers of these outcomes.

Medicaid—and other payers—rarely allocate resources to mitigate telehealth disparities, such as internet access, equipment (eg, tablets), or training.^33^ Consequently, vulnerable populations, such as older adults, patients with low socioeconomic status, and those with limited English proficiency, face continued challenges accessing telehealth.^45,46,47,48^ Our results extend prior findings around telehealth barriers by highlighting the efforts put forth by FQHCs to obtain funds to address telehealth disparities and demonstrate they experience mixed results. To overcome these gaps, reliable long-term investment is needed at the federal and state level, including access to broadband (eg, in line with the Federal Infrastructure Bill) and patient training for telehealth navigation, as suggested by some participants.^49^

We also found that there are divergent modality preferences within and between FQHC practitioners and patients.^50,51^ Importantly, interviewed practitioners expressed caution about relying on telehealth rather than physically examining patients. Still, our findings suggest that FQHCs leadership support flexibility and hybrid options. As FQHCs strive to adapt to the shifts in workforce expectations around compensation and modalities, future studies should examine how to create strategies that promote patient-centered care while also developing desirable working conditions in this competitive health care marketplace.

### Limitations

This study has several limitations that must be considered. Namely, the study findings are not necessarily generalizable, given the qualitative nature of this study and the relatively small sample size. Because of this study’s focus on CHCs, study participants solely represent the perspectives of individuals within these institutions, and do not include other policymakers. In addition, the CHCs selected for this study are in New York, New York, and thus may not represent countrywide geographic and population variation. Nonetheless, the results of this study serve as a basis for further examination of these themes on a broader scale, as well as identification of geographic and other variations in findings.

## Conclusions

In this qualitative study, we found that FQHC leadership and staff perceived telehealth Medicaid reimbursement policies in New York State as a factor that exacerbates inequities to access care, particularly for mental health needs. FQHCs staff and leadership reported opportunities to improve compliance, no-shows, and workflows through telehealth, but improvements in funding policy such as payment parity and more grants that can be used to address telehealth infrastructure (eg, Internet access, equipment, and literacy) are urgently needed.

## Notes

Supplement.
Data Sharing Statement
