# Supplementary material for: Experiences of Telehealth Reimbursement Policies in Federally Qualified Health Centers
Source: JAMA Netw Open. 2025 Feb 12;8(2):e2459554. doi: 10.1001/jamanetworkopen.2024.59554 (PMC11822543; doi:10.1001/jamanetworkopen.2024.59554)
Supplement: Supplement. — Data Sharing Statement [file jamanetwopen-e2459554-s001.pdf]

Data Sharing Statement

Porteny. Experiences of Telehealth Reimbursement Policies in Federally Qualified Health Centers. *JAMA Netw Open*. Published February 12, 2025.
doi:10.1001/jamanetworkopen.2024.59554

Data
Data available: No

Additional Information
Explanation for why data not available: We are committed to protecting the privacy and confidentiality of our participants. Data from this study is not publicly available for replication purposes. Our qualitative study contains deidentified data, but due to the sample size and sensitive nature of the interviews, there is still a possibility they could be identified by the details of their experiences.
